# Supplementary material for: Content-rich biological network constructed by mining PubMed abstracts
Source: BMC Bioinformatics. 2004 Oct 8;5:147. doi: 10.1186/1471-2105-5-147 (PMC528731; doi:10.1186/1471-2105-5-147)
Supplement: Additional File 2 — The original results of the above study (non-essential files are deleted to keep the file size under the limit set by BMC bioinformatics). [file 1471-2105-5-147-S2.bz2 › chilibotAdditionalFile2/dip30/465ID9660793E10492/html/CXCR3_CXCL10.html]

 


 **CXCR3** and **CXCL10** 
  
Found 773 abstracts in PubMed, retrieved 30.  
 

 What does Google say? 
 PDF only 
| .edu only 

---

**Interactive relationship** (e.g. stimulation, inhibition, etc)

**Neutral relationship**- These findings suggest that endometrial IP 10  [ **CXCL10** ]   [ **CXCR3** ]  regulates the establishment of apical interactions between trophoblast and epithelial cells during early gestation.  Ref: 12756249 J Biol Chem< MedlineTA>J Biol Chem,
- Human microvascular endothelial cell line 1 HMEC 1, transfected with either the known  **CXCR3**  renamed  **CXCR3**  A or  **CXCR3**  B, bound CXCL9,  **CXCL10** , and CXCL11.  Ref: 12782716 J Exp Med, 2003
- The chemokine receptor  **CXCR3**  is predominantly expressed on T lymphocytes, and its agonists CXCL9,  **CXCL10**  and CXCL11 are IFN gamma inducible chemokines that promote Th1 responses.  Ref: 12884299 Eur J Immunol, 2003
- The present findings suggest that MCP 1, RANTES, and IP 10  [ **CXCL10** ]   [ **CXCR3** ]  may participate in the pathogenesis of mercuric chloride induced tubulointerstitial fibrosis with mononuclear cell infiltration, via CCR2, CCR1 or CCR5, and  **CXCR3** , respectively.  Ref: 12834626 Exp Mol Pathol, 2003
- An Alternatively Spliced Variant of  **CXCR3**  Mediates the Inhibition of Endothelial Cell Growth Induced by IP 10  [ **CXCL10** ]   [ **CXCR3** ] , Mig, and I TAC, and Acts as Functional Receptor for Platelet Factor 4.  Ref: 12782716 J Exp Med, 2003
- Furthermore, a statistically significant increase in serum  **CXCL10**  IP 10  [ **CXCL10** ]   [ **CXCR3** ]  levels has been found in newly diagnosed Graves patients when compared to healthy subjects as well as patients with long standing disease and a strong statistically significant inverse correlation between circulating  **CXCL10**  IP 10  [ **CXCL10** ]   [ **CXCR3** ]  levels and disease duration has been demonstrated.  Ref: 12739748 J Endocrinol Invest, 2003

**Non-interactive relationship** (e.g. studied together, co-existance, homology, etc.)

- Correspondingly, the rate or intensity of inflammation in IL 10 mice treated with anti IP 10  [ **CXCL10** ]   [ **CXCR3** ]  Abs showed improved scoring of inflammation, compared with control IL 10 mice.  Ref: 12874231 J Immunol, 2003
- Assessment of cytokine production at the tumor site showed an interdependence of IFNgamma, MIG CXCL9 and IP 10  [ **CXCL10** ]   [ **CXCR3** ]   **CXCL10** .  Ref: 12740040 Mol Cancer< MedlineTA>Mol Cancer, 2003
- Expression of the chemokine IP 10  [ **CXCL10** ]   [ **CXCR3** ]  correlates with the accumulation of hepatic IFN gamma and IL 18 mRNA in chronic hepatitis C but not in hepatitis B.  Ref: 12794718 J Med Virol, 2003
- This study provides important and novel information regarding IP 10  [ **CXCL10** ]   [ **CXCR3** ]  as a target for the treatment of colitis.  Ref: 12874231 J Immunol, 2003
- Analytical ultracentrifugation indicates that, in free solution, IP 10  [ **CXCL10** ]   [ **CXCR3** ]  exists in a monomer dimer equilibrium with a dissociation constant of 9 microM.  Ref: 12737818 Structure Camb , 2003
- Potential molecular differences between early and late rejection grade > =2 episodes were examined by testing chemokine and receptor gene expression.METHODS Expression of inducible protein IP 10  [ **CXCL10** ]   [ **CXCR3** ] , monokine induced by IFN gamma Mig, interferon inducible T cell alpha chemoattractant I TAC, regulated on activation normal T cell expressed and secreted RANTES, and their receptors  **CXCR3**  and CCR5 was tested in 60 endomyocardial biopsies from 24 patients using quantitative Taqman real time polymerase chain reaction PCR.  Ref: 12829909 Transplantation, 2003
- RESULTS We assessed the importance of IFNgamma, IP 10  [ **CXCL10** ]   [ **CXCR3** ]   **CXCL10**  and MIG CXCL9 in SLC CCL21 therapy.  Ref: 12740040 Mol Cancer< MedlineTA>Mol Cancer, 2003
- The chemokines CXCL9 Mig,  **CXCL10**  IP 10  [ **CXCL10** ]   [ **CXCR3** ] , and CXCL11 I TAC regulate lymphocyte chemotaxis, mediate vascular pericyte proliferation, and act as angiostatic agents, thus inhibiting tumor growth.  Ref: 12782716 J Exp Med, 2003
- In addition, IL 12 inhibits tumor angiogenesis mainly through IFNgamma dependent production of the chemokine IP10  [ **CXCL10** ]   [ **CXCR3** ] .  Ref: 12871184 Curr Pharm Des, 2003
- I measured VEGF and IP 10  [ **CXCL10** ]   [ **CXCR3** ]  in normal human breast tissue in situ in healthy women, using microdialysis, in the follicular and luteal phase of the menstrual cycle.  Ref: 12788875 J Clin Endocrinol Metab, 2003
- Consistent with these results, infected cells from WT mice preferentially migrated in response to IP 10  [ **CXCL10** ]   [ **CXCR3** ]   **CXCR3**  ligand, while those from GKO mice migrated in response to eotaxin CCL11 CCR3 ligand.  Ref: 12875978 Am J Pathol, 2003
- Hepatic inflammatory activity was found to be associated more closely with IFN gamma than with IP 10  [ **CXCL10** ]   [ **CXCR3** ]  or IL 18 mRNA expression.  Ref: 12794718 J Med Virol, 2003
- DESIGN AND PATIENTS Immunocytochemistry and reverse transcriptase polymerase chain reaction RT PCR amplification were used to analyse the expression of monocyte chemoattractant protein MCP 1, RANTES regulated upon activation, normal T cell expressed and secreted, macrophage inflammatory protein MIP 1alpha, MIP 1beta, interferon IFN gamma inducible protein IP 10  [ **CXCL10** ]   [ **CXCR3** ]  and monokine induced by IFN gamma Mig in thyroid tissue from patients with Hashimoto s thyroiditis n = 4, Graves disease n = 6 and nonautoimmune multinodular goitre n = 4.  Ref: 12864798 Clin Endocrinol (Oxf), 2003
- Interferon gamma inducible chemokines CXCL9 Mig,  **CXCL10**  IP 10  [ **CXCL10** ]   [ **CXCR3** ] , CXCL11 I TAC, strongly associated to Th1 mediated immune responses, belong to the CXC sub family.  Ref: 12739748 J Endocrinol Invest, 2003
- Infiltrating CD8 T Cells in Oral Lichen Planus Predominantly Express CCR5 and  **CXCR3**  and Carry Respective Chemokine Ligands RANTES CCL5 and IP 10  [ **CXCL10** ]   [ **CXCR3** ]   **CXCL10**  in Their Cytolytic Granules A Potential Self Recruiting Mechanism.  Ref: 12819030 Am J Pathol, 2003
- Remarkably, primary cultures of human microvascular endothelial cells, whose growth is inhibited by CXCL9,  **CXCL10** , CXCL11, and CXCL4, expressed  **CXCR3**  B, but not  **CXCR3**  A.  Ref: 12782716 J Exp Med, 2003
- We investigated the potential down regulators of chemokine secretion IL 4, IL 10, IL 13, dexamethasone, and PGE2, IL 10 and IL 13 down regulate M. tuberculosis induced IL 8, IP 10  [ **CXCL10** ]   [ **CXCR3** ] , RANTES, and MCP 1 secretion from MG 63 cells by between 48 and 94% P <.  Ref: 12797540 J Clin Immunol, 2003
- Regulation of blastocyst migration, apposition and initial adhesion by a chemokine, IFN gamma inducible protein 10 kDa IP 10  [ **CXCL10** ]   [ **CXCR3** ] , during early gestation.  Ref: 12756249 J Biol Chem< MedlineTA>J Biol Chem, 2003
- In the present study, expression of IP 10  [ **CXCL10** ]   [ **CXCR3** ]  was investigated both in serum and in the liver of patients with chronic hepatitis C and hepatitis B.  Ref: 12794718 J Med Virol, 2003
- Kupffer cell blockade significantly reduced liver TNF alpha, MIP 2, and IP 10  [ **CXCL10** ]   [ **CXCR3** ]  gene expression and liver inflammation after the administration of AdL.PB\* or AdL.F\*PB\*.  Ref: 12804145 Hum Gene Ther, 2003
- Immunofluorescent analysis revealed that IP 10  [ **CXCL10** ]   [ **CXCR3** ]  receptor,  **CXCR3** , was localized in the trophoblast cells, to which biotinylated recombinant caprine IP 10  [ **CXCL10** ]   [ **CXCR3** ]  rcIP 10 bound, suggesting that  **CXCR3**  receptor expressed in the trophoblast cells is functioning.  Ref: 12756249 J Biol Chem< MedlineTA>J Biol Chem, 2003
- The binding to IP 10  [ **CXCL10** ]   [ **CXCR3** ]  could be inhibited by SLC but not by a non mac25 AGM binding chemokine, EBI1 ligand chemokine ELC.  Ref: 12847218 J Immunol, 2003
- 2 Interferon IFN inducible anti viral proteins, including IFIT1, OAS1 and Mx1, and Th1 chemoattractant IP 10  [ **CXCL10** ]   [ **CXCR3** ] , were up regulated predominantly 6 to 24 hours after stimulation.  Ref: 12887736 BMC ImmunolBMC Immunol, 2003
- Expression of IL 8, MCP 1, MCP 3, and IP 10  [ **CXCL10** ]   [ **CXCR3** ]  was significantly higher in pouchitis than normal pouch.  Ref: 12827410 Int J Colorectal Dis< MedlineTA>Int J Colorectal Dis, 2003
- SLC CCL21 mediated anti tumor responses require IFNgamma, MIG CXCL9 and IP 10  [ **CXCL10** ]   [ **CXCR3** ]   **CXCL10** .  Ref: 12740040 Mol Cancer< MedlineTA>Mol Cancer, 2003
- Interferon inducible protein 10  [ **CXCL10** ]  IP 10  [ **CXCL10** ]   [ **CXCR3** ]  is a potent inhibitor of angiogenesis in vivo.  Ref: 12788875 J Clin Endocrinol Metab, 2003
- CCL21, IFN gamma inducible protein 10 IP 10  [ **CXCL10** ]   [ **CXCR3** ] .  Ref: 12847218 J Immunol, 2003
- IP 10  [ **CXCL10** ]   [ **CXCR3** ]  expression was highest in hepatitis C.  Ref: 12794718 J Med Virol, 2003
- This review discusses chemokine responses in relation to HSV infection of the cornea emphasizing the role of  **CXCR3**  chemokine signaling by the IFN gamma inducible ligands MIG, IP10  [ **CXCL10** ]   [ **CXCR3** ]  and I TAC and includes discussion of their potential role in immunopathology in the nervous system.  Ref: 12815542 Curr Eye Res, 2003
- The data support the hypothesis that IP 10  [ **CXCL10** ]   [ **CXCR3** ]  is responsible for the recruitment of Th cells and monocytes in chronic hepatitis C, and suggest that its role in chronic hepatitis B is less determining.  Ref: 12794718 J Med Virol, 2003
- There were significant correlations between MIG and interferon gamma IFN gamma or IP 10  [ **CXCL10** ]   [ **CXCR3** ]  levels p <.  Ref: 12856973 Acta Paediatr, 2003
- The binding sites for several IP 10  [ **CXCL10** ]   [ **CXCR3** ]  neutralizing mAbs have also been mapped.  Ref: 12737818 Structure Camb , 2003
- Lesional keratinocytes also overexpressed the ligands of  **CXCR3** , namely, MIG CXCL9,  **CXCL10** , and I TAC CXCL11.  Ref: 12819030 Am J Pathol, 2003
- The expression of MCP 1, MCP 3 and IP 10  [ **CXCL10** ]   [ **CXCR3** ]  were significantly lower after antibiotic treatment.  Ref: 12827410 Int J Colorectal Dis< MedlineTA>Int J Colorectal Dis, 2003
- IP 10  [ **CXCL10** ]   [ **CXCR3** ]  and MIG protein expression in mammary gland tissues was analysed by immunohistochemistry.  Ref: 12856973 Acta Paediatr, 2003
- Furthermore, the presence of CCL5 and  **CXCL10**  in the cytolytic granules of tissue infiltrating CD8 T cells expressing CCR5 and  **CXCR3**  reveals a potential self recruiting mechanism involving activated effector cytotoxic T cells.  Ref: 12819030 Am J Pathol, 2003
- However, 2 of 5 cases of HL like ATLL with EBV infection and 2 of 2 HL NS with EBV had IP10  [ **CXCL10** ]   [ **CXCR3** ]  MIG positive H& ; RS cells.  Ref: 12866030 Int J CancerInt J Cancer, 2003
- Upon binding with mac25 AGM, SLC and IP 10  [ **CXCL10** ]   [ **CXCR3** ]  retained all their Ca 2 signaling activity in vitro, suggesting that mac25 AGM can hold and present chemokines in the basal lamina of HEVs.  Ref: 12847218 J Immunol, 2003
- RESULTS Significant amounts of IP 10  [ **CXCL10** ]   [ **CXCR3** ]  and MIG were detected in human milk.  Ref: 12856973 Acta Paediatr, 2003
- Recombinant murine interferon gamma rmIFN gamma inhibited the growth of EMT6 cells, but not K1735 cells in vitro, and strongly induced the expression of the antiangiogenic chemokine interferon inducible protein 10  [ **CXCL10** ]  IP 10  [ **CXCL10** ]   [ **CXCR3** ]  by both cell lines.  Ref: 12866028 Int J Cancer, 2003
- Tumor necrosis factor alpha TNF alpha, macrophage inflammatory protein 2 MIP 2, and interferon gamma inducible protein 10  [ **CXCL10** ]  IP 10  [ **CXCL10** ]   [ **CXCR3** ]  genes were upregulated, with two distinct peaks of mRNA expression occurring at 6 hr and 5 days.  Ref: 12804145 Hum Gene Ther, 2003
- Through its effect in the IFNg induced Jak STAT1 pathway, VIP and PACAP are able to control the gene expression of IP 10  [ **CXCL10** ]   [ **CXCR3** ] , CD40 and iNOS, three microglia derived mediators that play an essential role in several pathologies, i.e. inflammation and autoimmune disorders.  Ref: 12754213 J Biol Chem< MedlineTA>J Biol Chem, 2003
- The data suggests that, in the luteal phase, VEGF and IP 10  [ **CXCL10** ]   [ **CXCR3** ] , in the normal human breast, exhibit a proangiogenic profile.  Ref: 12788875 J Clin Endocrinol Metab, 2003
- An involvement of the interferon gamma inducible chemokine IP 10  [ **CXCL10** ]   [ **CXCR3** ] , which is chemoattractive for stimulated Th1 cells and monocytes, is also suggested.  Ref: 12794718 J Med Virol, 2003
- The concentrations of IP 10  [ **CXCL10** ]   [ **CXCR3** ]  and MIG in colostrum and early milk were significantly higher than those of sera from healthy controls or lactating mothers.  Ref: 12856973 Acta Paediatr, 2003
- AIM To assess the immunological role of human milk by analysing the concentrations of interferon gamma inducible protein of 10 kda IP 10  [ **CXCL10** ]   [ **CXCR3** ]  and monokine induced by interferon gamma MIG in human milk from mothers of preterm and term infants.  Ref: 12856973 Acta Paediatr, 2003
- Chemotaxis assay indicated that rcIP 10 stimulated the migration of trophoblast cells, and the effects of rcIP 10 were neutralized by the pretreatment with an anti IP 10  [ **CXCL10** ]   [ **CXCR3** ]  antibody.  Ref: 12756249 J Biol Chem< MedlineTA>J Biol Chem, 2003
- IP 10  [ **CXCL10** ]   [ **CXCR3** ]  and MIG mRNA expression levels in cellular components of human milk were determined by RT PCR.  Ref: 12856973 Acta Paediatr, 2003
- Adhesion assay revealed that adhesive activity of trophoblast cells to fibronectin was promoted by rcIP 10, and the effect was inhibited by the use of anti IP 10  [ **CXCL10** ]   [ **CXCR3** ]  antibody.  Ref: 12756249 J Biol Chem< MedlineTA>J Biol Chem, 2003
- Interferon gamma inducible protein 10  [ **CXCL10** ]  IP10  [ **CXCL10** ]   [ **CXCR3** ]  and monokine induced by interferon gamma MIG positive H& ; RS cells were detected in all 10 HL MC but only in 5 of 10 cases of HL NS and 2 of 9 cases of HL like ATLL.  Ref: 12866030 Int J CancerInt J Cancer, 2003
- We have determined the structure of wild type IP 10  [ **CXCL10** ]   [ **CXCR3** ]  from three crystal forms.  Ref: 12737818 Structure Camb , 2003
- CCR3 functional responses are regulated by both  **CXCR3**  and its ligands CXCL9,  **CXCL10**  and CXCL11.  Ref: 12884299 Eur J Immunol, 2003
- The crystals provide eight separate models of the IP 10  [ **CXCL10** ]   [ **CXCR3** ]  chain, all differing substantially from a monomeric IP 10  [ **CXCL10** ]   [ **CXCR3** ]  variant examined previously by NMR spectroscopy.  Ref: 12737818 Structure Camb , 2003
- In each crystal form, IP 10  [ **CXCL10** ]   [ **CXCR3** ]  chains form conventional beta sheet dimers, which, in turn, form a distinct tetrameric assembly.  Ref: 12737818 Structure Camb , 2003
- Crystal structures of oligomeric forms of the IP 10  [ **CXCL10** ]   [ **CXCR3** ]   **CXCL10**  chemokine.  Ref: 12737818 Structure Camb , 2003
- CONCLUSION We postulate that TFC may play a role in the pathogenesis of autoimmune thyroid disease as they are able to express the chemokines MIP 1alpha, MIP 1beta, MCP 1, RANTES, IP 10  [ **CXCL10** ]   [ **CXCR3** ]  and Mig that would promote the infiltration of immune cells into the thyroid gland.  Ref: 12864798 Clin Endocrinol (Oxf), 2003
- IP 10  [ **CXCL10** ]   [ **CXCR3** ]  levels did not vary during the menstrual cycle either in breast tissue 65 17 pg ml vs. 75 21 pg ml, P = 0.6 or in plasma 64 7 pg ml vs. 81 10 pg ml, P = 0.06.  Ref: 12788875 J Clin Endocrinol Metab, 2003
- Inhibitory effect on CD40, IP 10  [ **CXCL10** ]   [ **CXCR3** ]  and iNOS expression.  Ref: 12754213 J Biol Chem< MedlineTA>J Biol Chem, 2003
- This work compares the proliferative, IgM, IL 6 and IP 10  [ **CXCL10** ]   [ **CXCR3** ]  response of PBMC from normal donors to a diverse panel of CpG ODNs.  Ref: 12869028 Clin Exp Immunol, 2003
- Specifically, IP 10  [ **CXCL10** ]   [ **CXCR3** ]  inhibition in IL 10 mice attenuates the associated increases in serum and or local amyloid A, IL 2, IL 6, TNF alpha, IFN gamma, IL 1alpha, and IL 1beta with colitis as compared with IL 10 mice that develop colitis similar to human Crohn s disease.  Ref: 12874231 J Immunol, 2003
- Furthermore, infiltrating T cells, particularly CD8 T cells, were positive for RANTES CCL5 and IP 10  [ **CXCL10** ]   [ **CXCR3** ]   **CXCL10** , the ligands of CCR5 and  **CXCR3** , respectively.  Ref: 12819030 Am J Pathol, 2003
- Biopsies were stained for CD68, CD3, elastase, eotaxin, IP 10  [ **CXCL10** ]   [ **CXCR3** ] , MCP 1, MCP 3, and IL 8 and analyzed by NIH Image analyzer.  Ref: 12827410 Int J Colorectal Dis< MedlineTA>Int J Colorectal Dis, 2003
- In chronic hepatitis C, but not in chronic hepatitis B nor in liver disorders unrelated to viral infections, IP 10  [ **CXCL10** ]   [ **CXCR3** ]  expression was strongly correlated with the amount of transcripts for IFN gamma and to the amount of transcripts for the constitutively expressed macrophage derived cytokine IL 18.  Ref: 12794718 J Med Virol, 2003
- Expression of IP 10  [ **CXCL10** ]   [ **CXCR3** ]  and MIG genes and proteins in the milk cells as well as in mammary gland epithelial tissues was detected by RT PCR and immunohistochemistry.  Ref: 12856973 Acta Paediatr, 2003
- A statistically significant increase of  **CXCL10**  IP 10  [ **CXCL10** ]   [ **CXCR3** ]  and CXCL9 Mig expression, in thyroid tissue specimens obtained from subjects affected by Hashimoto s thyroiditis and recent onset Graves disease has been reported.  Ref: 12739748 J Endocrinol Invest, 2003
- CONCLUSION These findings indicate that the full potency of SLC CCL21 mediated anti tumor responses require in part the induction of IFNgamma, MIG CXCL9 and IP 10  [ **CXCL10** ]   [ **CXCR3** ]   **CXCL10** .  Ref: 12740040 Mol Cancer< MedlineTA>Mol Cancer, 2003
- The present study investigated the molecular mechanisms involved in the VIP PACAP regulation of several IFNg induced microglia derived factors, including IFNg inducible protein 10 IP 10  [ **CXCL10** ]   [ **CXCR3** ] , inducible nitric oxide synthase iNOS and CD40.  Ref: 12754213 J Biol Chem< MedlineTA>J Biol Chem, 2003
- Using an HBV transgenic mouse model, a reduction of hepatic infiltration and liver disease was achieved recently by administration of antibodies directed against the interferon gamma inducible chemokine Mig and against IP 10  [ **CXCL10** ]   [ **CXCR3** ] .  Ref: 12794718 J Med Virol, 2003
- Our findings show that IP 10  [ **CXCL10** ]   [ **CXCR3** ]  significantly contributes to the development of Th1 and inflammatory responses.  Ref: 12874231 J Immunol, 2003
- The binding of mac25 AGM to SLC and IP 10  [ **CXCL10** ]   [ **CXCR3** ]  was dose dependent and saturable.  Ref: 12847218 J Immunol, 2003
- Real time, quantitative reverse transcriptase polymerase chain reaction analysis demonstrates that the  **CXCR3**  ligands, interferon IFN inducible protein 10  **CXCL10**  and IFN inducible T cell alpha chemoattractant CXCL11, are among the many chemokines induced in the adjuvant treated peritoneum.  Ref: 12773510 J Leukoc Biol, 2003
- Immunohistochemical analysis indicated that mac25 AGM colocalizes at least partially with SLC and IP 10  [ **CXCL10** ]   [ **CXCR3** ]  at the basal lamina of HEVs.  Ref: 12847218 J Immunol, 2003
- In contrast, IL 4 augments gene expression and secretion of IP 10  [ **CXCL10** ]   [ **CXCR3** ]  from 1 30 1070 to 2 30 1720 pg ml 4 x 10 5 cells P <.  Ref: 12797540 J Clin Immunol, 2003
- CONCLUSION IP 10  [ **CXCL10** ]   [ **CXCR3** ]  and MIG in human milk, probably derived from milk cells and mammary gland epithelial cells, may contribute to the migration and activation of intestinal T lymphocytes to enhance mucosal immunity during the early neonatal period.  Ref: 12856973 Acta Paediatr, 2003
- We have previously shown that SLC CCL21 mediated anti tumor responses are accompanied by significant induction of IFNgamma and the CXC chemokines, monokine induced by IFNgamma MIG CXCL9 and IFNgamma inducible protein 10 IP 10  [ **CXCL10** ]   [ **CXCR3** ]   **CXCL10** .  Ref: 12740040 Mol Cancer< MedlineTA>Mol Cancer, 2003
- RESULTS Expression of MCP 1, RANTES, MIP 1alpha, MIP 1beta, IP 10  [ **CXCL10** ]   [ **CXCR3** ]  and Mig was demonstrated in all Hashimoto s and most Graves thyroid specimens but very little expression was detected in the nonautoimmune goitre samples.  Ref: 12864798 Clin Endocrinol (Oxf), 2003
- In the lesions, the expression of MCP 1 mRNA was most prominently elevated, and those of RANTES and IP 10  [ **CXCL10** ]   [ **CXCR3** ]  mRNAs also increased, and their proteins were localized in tubular epithelium.  Ref: 12834626 Exp Mol Pathol, 2003
- METHODS IP 10  [ **CXCL10** ]   [ **CXCR3** ]  and MIG levels of colostrum, early milk, mature milk and sera were measured by enzyme linked immunosorbent assay ELISA.  Ref: 12856973 Acta Paediatr, 2003
- High levels of RANTES CCL5, MCP 1 CCL2, IP 10  [ **CXCL10** ]   [ **CXCR3** ]   **CXCL10** , and Mig CXCL9 simultaneously with mononuclear cell infiltration in the lungs was found.  Ref: 12875978 Am J Pathol, 2003
- In vivo depletion of IP 10  [ **CXCL10** ]   [ **CXCR3** ]   **CXCL10** , MIG CXCL9 or IFNgamma significantly reduced the anti tumor efficacy of SLC CCL21.  Ref: 12740040 Mol Cancer< MedlineTA>Mol Cancer, 2003
- We first studied endometrial IP 10  [ **CXCL10** ]   [ **CXCR3** ]  mRNA expression, which was localized in the subepithelial stromal region, and detected the protein in the uterine flushing media during early pregnancy.  Ref: 12756249 J Biol Chem< MedlineTA>J Biol Chem, 2003
- Neuronal and astrocytic apoptosis in these cultures was significantly inhibited by co treatment with MCP 1 or RANTES but not IP 10  [ **CXCL10** ]   [ **CXCR3** ] .  Ref: 12753088 J Neurochem, 2003
- The results indicate that VIP PACAP inhibit Jak1 2 and STAT1 phosphorylation, and the binding of activated STAT1 to the IFNg activated site GAS motif in the IFN regulatory factor 1 IRF 1 and CD40 promoter and to the IFN stimulated response element ISRE motif of the IP 10  [ **CXCL10** ]   [ **CXCR3** ]  promoter.  Ref: 12754213 J Biol Chem< MedlineTA>J Biol Chem, 2003
- In this study, we examined the effect of an endometrial chemokine, interferon gamma inducible protein 10  [ **CXCL10** ]  kDa IP 10  [ **CXCL10** ]   [ **CXCR3** ] , on conceptus migration to the endometrial epithelium.  Ref: 12756249 J Biol Chem< MedlineTA>J Biol Chem, 2003
- Expression of IP 10  [ **CXCL10** ]   [ **CXCR3** ]  mRNA by the endometrium of cyclic animals was stimulated by the addition of a conceptus factor interferon tau IFN tau.  Ref: 12756249 J Biol Chem< MedlineTA>J Biol Chem, 2003
- The biopsies were taken in the first 3 months or from the 9th to the 12th month following transplantation.RESULTS IP 10  [ **CXCL10** ]   [ **CXCR3** ] , Mig, RANTES,  **CXCR3** , and CCR5 expression levels were increased in the later versus earlier biopsies P< ; =0.01 despite no change in histologic rejection grade status.CONCLUSION These results demonstrate significantly increased expression of T cell chemoattractants in heart allografts during later rejection when compared with episodes occurring shortly after transplantation.  Ref: 12829909 Transplantation, 2003
- IFN gamma inducible protein 10 IP 10  [ **CXCL10** ]   [ **CXCR3** ]  and  **CXCR3**  are highly expressed at sites of colitis.  Ref: 12874231 J Immunol, 2003
